# Combined hamartoma of the retina and retinal pigment epithelium – MRI features of a rare paediatric intraocular tumour

**DOI:** 10.1259/bjrcr.20200077

**Published:** 2020-11-17

**Authors:** Stephan Waelti, Tim Fischer, Veit Sturm, Jan Heckmann

**Affiliations:** 1 Department of Radiology and Nuclear Medicine, Children’s Hospital of Eastern Switzerland, Claudiusstrasse 6, 9006 St. Gallen, Switzerland; 2 Department of Radiology and Nuclear Medicine, Kantonsspital St. Gallen, Rorschacher Strasse 95, 9007 St. Gallen, Switzerland; 3 Department of Ophthalmology, Kantonsspital St. Gallen, Rorschacher Strasse 95, 9007 St. Gallen, Switzerland

## Abstract

Combined hamartoma of the retina and retinal pigment epithelium is a rare benign ocular tumour in children, composed of glial cells, vascular tissue, and sheets of pigmented epithelial cells. Although generally thought to be congenital, acquired cases are known to exist. It usually presents with reduced visual acuity and/or strabismus and it can be associated with several syndromes, including Neurofibromatosis Type 2. There is no consensus on the management of combined hamartoma of the retina and retinal pigment epithelium.

We present a case, including MRI features, of a 4,5-years-old girl with a combined hamartoma of the retina and retinal pigment epithelium.

## Clinical presentation

A 4,5-years-old girl was referred by her paediatrician for a brain MRI because of a new ocular motility disorder affecting her right eye. The girl has already been examined ophthalmologically from an early age because of a relative amblyopia of her right eye. At that time, she was diagnosed with Bergmeister’s papilla. Bergmeister’s papilla is a harmless variant of the papilla formed by a posterior remnant of the hyaloid artery presenting as an epipapillary membrane. The patient was otherwise healthy.

## Investigations/Imaging findings

**Figure 1.**
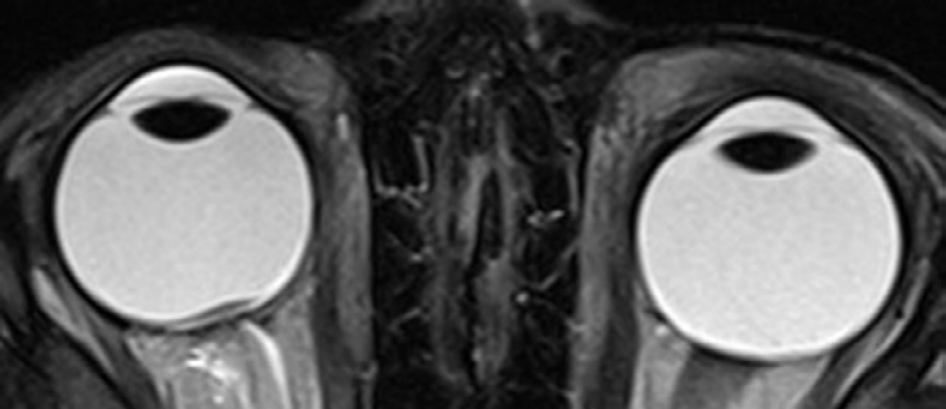
*T*_2_W axial image showing a plaque-shaped lesion immediately in front of the papilla of the right eye. The lesion is wider than the width of the papilla. Note the thin, hyperintense layer between the lesion and the papilla being the normal choroid underneath the affected retina. The optic nerves are normal. The apparent asymmetry is due to the inexact transversal orientation of the plane.

**Figure 2.**
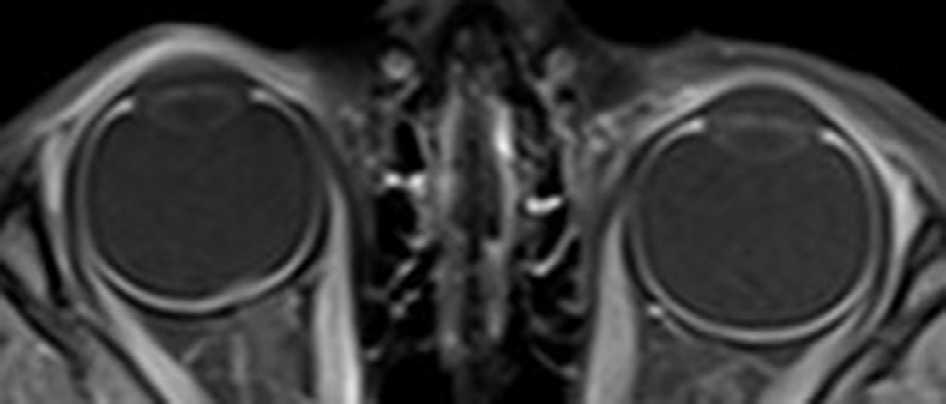
*T*_1_W fs with Gadolinium axial image showing a discrete enhancement of the lesion.

An MRI with a specific orbital protocol was conducted (thin-slice coronal *T*_1_W and *T*_2_W fs, axial *T*_2_W, coronal and axial post-contrast *T*_1_W fs). It revealed a plaque-shaped lesion immediately in front of the papilla of the right eye. The lesion was about 6 mm wide, wider than the width of the papilla, and less than 1 mm thick (Figure 1). On *T*_1_W and *T*_2_W images it was almost isointense to the optic nerve. On *T*_1_W images using fat saturation and contrast agent, a discrete enhancement could be assumed (Figure 2). Between the lesion and the papilla, there was a very thin layer with isointense signal on *T*_2_W images to the vitreous body and enhancement being the normal choroid underneath the affected retina. There were no signs of calcifications. The eye size was normal compared to the contralateral eye. The rest of the right eye, the entire left eye, the optic nerves, and the rest of the brain MRI were normal. No explanation for the eye motility disorder could be found.

**Figure 3.**
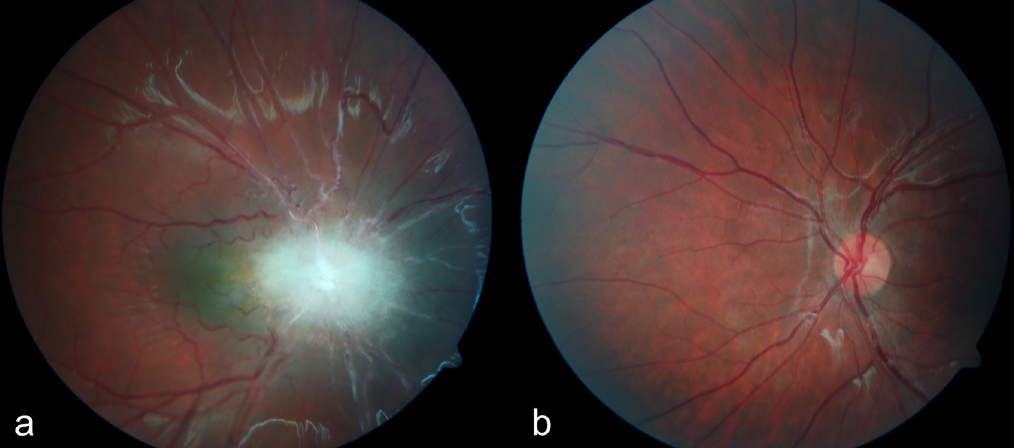
(a) Fundoscopy, right eye: Ill-defined, greyish retinal mass on the optic nerve, extending to the peripapillary retina, with a hyperpigmented margin and some obscuration of the major vessels, as well as vessel tortuosity. (b) Fundoscopy, normal left eye: Easily distinguishable papilla and normal vessels.

After the MRI, an ophthalmological examination, including fundoscopy and optical coherence tomography, was carried out by a paediatric ophthalmologist, and the diagnosis of a combined hamartoma of the retina and retinal pigment epithelium (CHR-RPE) was made (Figure 3a and b).

## Differential diagnosis

The radiological differential diagnosis of unilateral papillary or peripapillary mass or disk swelling in the first decade of life is broad and also includes the pathologies mentioned below:

- Optic nerve drusen: they are limited to the papilla and can calcify.
- Neoplasia: it is important to distinguish the benign CHR-RPE from malignant chorioretinal lesions, in particular retinoblastoma and choroidal melanoma, as misdiagnoses could lead to unnecessary enucleations. Retinoblastoma and melanoma are usually mass-like lesions and not plaque-shaped lesions and show contrast enhancement. Retinoblastoma generally shows calcifications in ultrasound.
- Infiltrative pathologies: leukaemia is a common cause of infiltrative disease in children. Look for an abnormal signal of the bone marrow. Neurosarcoidosis presenting with optic nerve infiltration is very uncommon in children. Look for basilar meningitis, and/or pulmonary disease.^1^
- Optic neuritis: optic neuritis in prepubescents is usually bilateral. The most commonly identified cause of neuroretinitis in the first decade of life is Bartonella (serological work-up).^1^
- Increased intracranial pressure: papilledema is usually bilateral. MRI can depict additional findings like widened perioptic spaces, flattened globe, or partially empty sella.

## Discussion

CHR-RPE is a rare, benign, ocular tumour of the retina and retinal pigment epithelium. It consists of a proliferation of glial and vascular elements in combination with sheets of pigmented epithelial cells.^2,3^ Punctate foci of calcification have been reported in longstanding disease. Variations in the relative proportions of these components contribute to a heterogeneous clinical appearance. While classically thought to be congenital, there are reports of acquired cases.^4^ It was first described in 1973.

CHR-RPE is almost always solitary and unilateral. It usually presents during childhood (median age 7.5 year) with reduced acuity (40%) and/or strabismus (28%).^5,6^ It can occur in the macula (17%), juxtapapillary (76%), or in the peripheral retina (7%).^7^ Visual acuity varies with the location of the lesion. When located in the macula, it can cause significant vision loss.^6^ The majority of CHR-RPE occurs sporadically. However, it has been reported to occur in association with neurofibromatosis Types 1 and 2, tuberous sclerosis, Gorlin syndrome, branchio-oculo-facial syndrome, nasopharyngeal angiofibroma, and Poland syndrome.^8–14^ One author has even recommended to screen children with CHR-RPE for NF–2.

An ophthalmologist with paediatric experience will be able to diagnose a CHR-RPE. Because of its rarity and variable clinical appearance, misdiagnoses are common and include melanoma, retinoblastoma, Bergmeister’s papilla (as in our case), congenital hypertrophy of the RPE, or vascular anomaly. Early stages of this tumour can mimic disk swelling.

The diagnosis cannot be made using radiological imaging alone. We believe that radiology is primarily useful to rule out other causes and to look for any findings of the above-mentioned associated syndromes that remain hidden from the ophthalmologist.

## Management

There is no established consensus on the management of CHR-RPE. Surgical interventions (vitrectomy and membrane peeling) can be considered when the child is of amblyopic age, or when the visual acuity declines significantly. A visual improvement rate of 60% has been reported after surgery. Unfortunately, the epiretinal membrane often recurs and subsequent surgery tends to be increasingly less effective due to permanent architectural changes.^15^ Our patient received no treatment. An ophthalmologic check-up is planned every 3 months due to the associated strabismus. At the last routine check-up in July 2020, there was no change in the fundoscopic findings.

## Learning points

- CHR-RPE is a rare benign ocular tumour in children.
- CHR-RPE can occur in association with several syndromes, including neurofibromatosis Type 2.
- On MRI, it seems to be a plaque-shaped lesion, unlike the more mass-like retinoblastoma and melanoma.
- CHR-RPE is almost always unilateral, unlike papilledema.

## References

[1] Mammo DA, Quiram PA, Lee MS, Siatkowski RM. A child with unilateral disk elevation. Surv Ophthalmol 2019;28 May 2019. doi: 10.1016/j.survophthal.2019.05.004 31150657

[2] Chawla R, Kumar V, Tripathy K, Kumar A, Venkatesh P, Shaikh F, et al. Combined hamartoma of the retina and retinal pigment epithelium: an optical coherence tomography-based reappraisal. Am J Ophthalmol 2017; 181: 88–96. doi: 10.1016/j.ajo.2017.06.020 28669779

[3] Xue K, Mellington F, Gout I, Rokerya S, Olurin OI, El-Amir A. Combined hamartoma of the retina and retinal pigment epithelium. BMJ Case Rep 2012; 2012: bcr2012006944: bcr2012006944 15 Nov 2012. doi: 10.1136/bcr-2012-006944 23162024 PMC4543858

[4] Yonekawa Y, Thomas BJ, Drenser KA, Trese MT, Capone A. Acquired combined hamartoma of the retina and retinal pigment epithelium. JAMA Ophthalmol 2015; 133: 1085–6. doi: 10.1001/jamaophthalmol.2015.1675 26043305

[5] Scupola A, Grimaldi G, Sammarco MG, Sasso P, Marullo M, Blasi MA. Multimodal imaging evaluation of combined hamartoma of the retina and retinal pigment epithelium. Eur J Ophthalmol 2020; 30: 595–9. doi: 10.1177/1120672119831223 30764657

[6] Shields CL, Thangappan A, Hartzell K, Valente P, Pirondini C, Shields JA. Combined hamartoma of the retina and retinal pigment epithelium in 77 consecutive patients visual outcome based on macular versus extramacular tumor location. Ophthalmology 2008; 115: 2246–52. doi: 10.1016/j.ophtha.2008.08.008 18995912

[7] Font RL, Moura RA, Shetlar DJ, Martinez JA, McPherson AR. Combined hamartoma of sensory retina and retinal pigment epithelium. Retina 1989; 9: 302–11. doi: 10.1097/00006982-198909040-00011 2697919

[8] Vianna RN, Pacheco DF, Vasconcelos MM, de Laey JJ. Combined hamartoma of the retina and retinal pigment epithelium associated with neurofibromatosis type-1. Int Ophthalmol 2001; 24: 63–6. doi: 10.1023/A:1016316114746 12201346

[9] Tsai P, O'Brien JM. Combined hamartoma of the retina and retinal pigment epithelium as the presenting sign of neurofibromatosis-1. Ophthalmic Surg Lasers 2000; 31: 145–7. 10743927

[10] Sivalingam A, Augsburger J, Perilongo G, Zimmerman R, Barabas G. Combined hamartoma of the retina and retinal pigment epithelium in a patient with neurofibromatosis type 2. J Pediatr Ophthalmol Strabismus 1991; 28: 320–2. 1757856 10.3928/0191-3913-19911101-08

[11] De Potter P, Stanescu D, Caspers-Velu L, Hofmans A. Photo essay: combined hamartoma of the retina and retinal pigment epithelium in Gorlin syndrome. Arch Ophthalmol 2000; 118: 1004–5. 10900122

[12] Demirci H, Shields CL, Shields JA. New ophthalmic manifestations of Branchio-Oculo-Facial syndrome. Am J Ophthalmol 2005; 139: 362–4. doi: 10.1016/j.ajo.2004.07.052 15734008

[13] Fonseca RA, Dantas MA, Kaga T, Spaide RF. Combined hamartoma of the retina and retinal pigment epithelium associated with juvenile nasopharyngeal angiofibroma. Am J Ophthalmol 2001; 132: 131–2. doi: 10.1016/S0002-9394(00)00952-1 11438076

[14] Stupp T, Pavlidis M, Bochner T, Thanos S. Poland anomaly associated with ipsilateral combined hamartoma of retina and retinal pigment epithelium. Eye 2004; 18: 550–2. doi: 10.1038/sj.eye.6700714 15131695

[15] Zhang X, Dong F, Dai R, Yu W. Surgical management of epiretinal membrane in combined hamartomas of the retina and retinal pigment epithelium. Retina 2010; 30: 305–9. 20175272

